# Effects of a warm compress containing menthol on the tear film in healthy subjects and dry eye patients

**DOI:** 10.1038/srep45848

**Published:** 2017-04-05

**Authors:** Reiko Arita, Naoyuki Morishige, Ichiro Sakamoto, Natsuko Imai, Yuko Shimada, Michihito Igaki, Atsushi Suzuki, Kouzo Itoh, Kazuo Tsubota

**Affiliations:** 1 Itoh Clinic, 626-11 Minami-Nakano, Minuma-ku, Saitama, Saitama 337-0042, Japan; 2 Department of Ophthalmology, Keio University, 35 Shinanomachi, Shinjyuku-ku, Tokyo 160-0016, Japan; 3 Lid and Meibomian Gland Working Group, 2-11-15-1401 Koishikawa, Bunkyo-ku, Tokyo 1120002, Japan; 4 Division of Cornea and Ocular Surface, Ohshima Eye Hospital, 11-8 Kami-Gofukumachi, Hakata-ku, Fukuoka, Fukuoka 812-0036, Japan; 5 Personal Health Care Products Research Laboratories, Kao Corporation, 2-1-3 Bunka, Sumida-ku, Tokyo 131-8501, Japan

## Abstract

Menthol is thought to stimulate lacrimation via activation of cold-sensitive primary afferent neurons in the cornea. We evaluated a warm compress containing menthol as a potential treatment for dry eye by examining its effects on the tear film in healthy subjects (*n* = 20) and dry eye patients (*n* = 35). Disposable eyelid-warming steamers that either did (MH) or did not (HO) contain menthol were applied to one eye of each subject either once only for 10 min or repeatedly over 2 weeks. Single application of MH significantly increased tear meniscus volume (*P* = 8.6 × 10^−5^, *P* = 1.3 × 10^−5^) and tear film breakup time (*P* = 0.006, *P* = 0.002) as well as improved meibum condition in healthy subjects and dry eye patients, respectively. Repeated application of MH significantly increased tear meniscus volume (*P* = 0.004, *P* = 1.7 × 10^−4^) and tear film breakup time (*P* = 0.037, *P* = 0.010) in healthy subjects and dry eye patients, respectively. Repeated application of MH thus induced persistent increases in tear fluid volume and tear film stability in dry eye patients, suggesting that repeated use of a warm compress containing menthol is a potential novel treatment for dry eye disease.

Menthol is a volatile water-clear organic compound with the structure of a cyclic monoterpene that is incorporated into various consumer products including toothpaste, gum, and medicines such as proprietary eyedrops. It has also been suggested as a possible treatment for dry eye^1^,^2^,^3^,^4^. Menthol binds to transient receptor potential cation channel subfamily M member 8 (TRPM8)^1^,^2^,^3^,^5^,^6^,^7^ and thereby stimulates lacrimation via activation of cold-sensitive primary afferent neurons in the cornea. The possible effects of menthol on tear volume and tear film stability in humans have remained unknown, however.

Dry eye is characterized by qualitative or quantitative abnormalities of the tear film and is estimated to affect and have a negative impact on the quality of life of ~4.88 million people in the United States^8^,^9^,^10^. If left untreated, dry eye can result in damage to the ocular surface and impair vision. A reduced volume of tear fluid gives rise to aqueous-deficient dry eye (ADDE), whereas impaired tear film stability underlies evaporative dry eye (EDE)^11^. ADDE is further categorized as Sjӧgren syndrome–type ADDE (SS-ADDE) or non-Sjӧgren ADDE (non-SS-ADDE). The common etiology of SS-ADDE and non-SS-ADDE is dysfunction or hypofunction of lacrimal glands and a consequent deficiency of the aqueous layer of the tear film^11^. Meibomian gland dysfunction (MGD), a chronic abnormality of meibomian glands characterized by terminal duct obstruction or qualitative or quantitative changes in glandular secretion^12^, is one of the most important underlying causes of EDE^13^. Meibomian glands produce meibum, the lipid components of which give rise to the surface lipid layer of the tear film. MGD can thus result in changes to the tear film, symptoms of eye irritation, clinically apparent inflammation, and ocular surface disease^12^.

Given the difference in the pathophysiologies of ADDE and EDE, the treatment strategies for these conditions are also different. ADDE is managed with punctal plugs and eyedrops such as those containing cyclosporine, artificial tears, hyaluronate, diquafosol sodium chloride, or rebamipide, with the aim of increasing the volume of tear fluid or tear film stability^14^. The application of a warm compress to the eyelids is a standard treatment for obstructive MGD^15^. Previous studies have assessed the effects of various warming devices—including infrared devices^16^,^17^, a disposable eyelid-warming device^18^, warm moist air devices^19^,^20^,^21^, an Orgahexa fiber eye mask^22^, Blephasteam^23^, MGDRx EyeBag^24^,^25^, Azuki-no-chikara^26^, and LipiFlow^27^—on tear function and the ocular surface.

The purpose of the present study was to investigate the effects of a disposable eyelid-warming steamer containing menthol (MH) on tear film function, meibomian glands, and the ocular surface in individuals with dry eye. We first examined the effects of a single application of MH and of a similar device without menthol (HO) in healthy subjects and dry eye patients. We then examined the effects of repeated warming with the two devices over a period of 2 weeks.

## Results

None of the subjects reported glare, discomfort, or pain during the examinations. Moreover, there were no reports of persistent complications as a result of the procedures.

### Single-use evaluation

**Figure 1:**
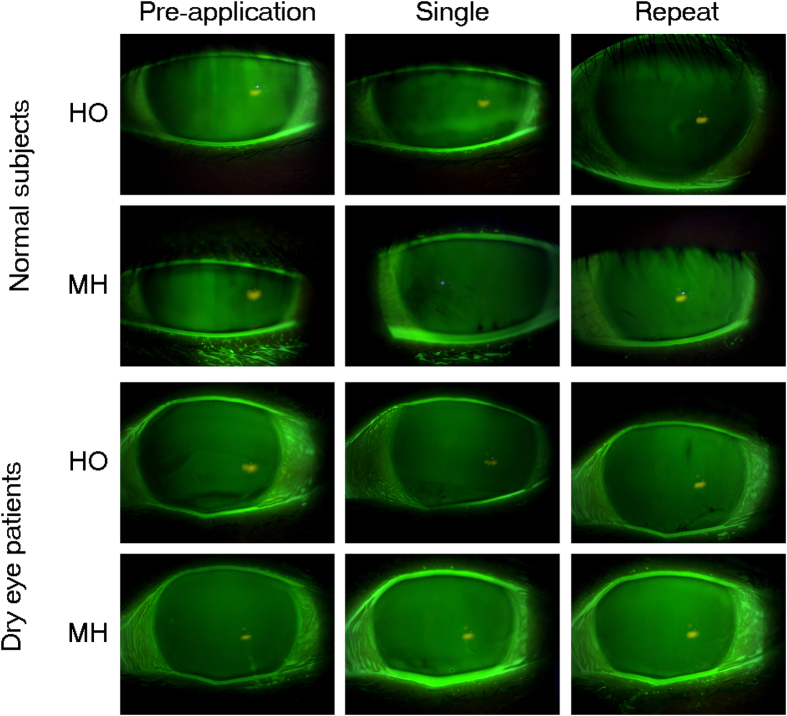
Slitlamp photographs of tear fluid before and after single application or repeat application of HO or MH eyelid-warming devices in normal subjects and dry eye patients. Note that only MH increased tear volume not only in normal subjects but also in dry eye patients.

Tear film parameters measured before and immediately after single application of MH (containing menthol) or HO (heat only) devices for 10 min are shown for all the subjects in Table 1. Tear meniscus volume (TMV) was significantly increased after MH application in both healthy subjects and dry eye patients, but it was not increased after HO application in either group. Tear film breakup time (BUT) was also significantly increased only after MH application in both groups. Meibum grade was significantly improved after application of MH or HO in both healthy subjects and dry eye patients. Fluorescein staining score as a measure of ocular surface damage was unaffected by either device in either group of participants. Representative images of the tear meniscus before and after single application of each device are shown in Fig. 1.

### Repeated-use evaluation

The Dry Eye–Related Quality-of-Life Score (DEQS) and tear film parameters measured before and at least 8 h after repeated application (10 min twice a day for 2 weeks) of HO or MH are shown for healthy subjects and dry eye patients in Table 2. DEQS was significantly improved after HO or MH application in dry eye patients but not in healthy subjects. TMV and tear film BUT were significantly increased after MH application, but not after HO application, in both groups of participants. Meibum grade was significantly improved after MH application in healthy subjects. Neither device significantly affected fluorescein staining score in either group of participants. Representative images of the tear meniscus before and after repeated application of HO or MH are shown in Fig. 1.

## Discussion

We have here shown that single application of a disposable eyelid-warming device containing menthol (MH) increased TMV and prolonged tear film BUT in both dry eye patients and healthy subjects, whereas single application of a similar device without menthol (HO) had no such effects. Furthermore, repeated application of MH, but not that of HO, induced persistent increases in TMV and BUT in dry eye patients as well as normal subjects. Our results thus indicate that single or repeated application of MH improved the condition of the tear film in both the normal and diseased state, suggesting that MH application is a potential novel treatment option for dry eye.

Single application of MH increased TMV and BUT as well as improved meibum condition, whereas HO improved meibum condition alone, in both healthy subjects and dry eye patients. Both menthol and cold temperatures are thought to increase tear secretion as a result of stimulation of TRPM8^1^,^2^,^3^,^4^,^5^,^6^. Cooling was thus previously shown to increase tear volume through TRPM8 signaling^3^, suggesting that warming might be expected to have a negative effect on the production of tear fluid. We have found that the application of eyedrops containing menthol or of chilled saline eyedrops did not increase TMV in healthy subjects (unpublished data). These observations, together with our present finding that only MH increased TMV in healthy subjects and dry eye patients, thus suggest that warming may be necessary for menthol to be able to increase tear fluid volume and that the regulation of TMV by menthol and temperature appears to be complex.

Repeated application of MH induced stable increases in both TMV and BUT in both normal subjects and dry eye patients. Given that tear film parameters were evaluated at least 8 h after the final application of MH, our results suggest that repeated MH use might give rise to a persistent stimulation of tear fluid production by lacrimal glands. The mechanisms of such persistent effects warrant further investigation, especially given that repeated application of menthol might be expected to result in desensitization of the TRPM8 response.

Given that menthol is a ligand of TRPM8, it might be expected to affect the sensory nervous system and thereby influence ocular symptoms. We found that repeated application of MH or HO for 2 weeks improved DEQS in dry eye patients. Although we did not evaluate the effect of repeated application of HO on ocular symptoms in healthy subjects, that of MH did not significantly affect DEQS in healthy subjects. Similar to our results, a recent study found that the application of low-dose menthol ointment improved the ocular symptom score in dry eye patients^7^; although the symptom score was worsened in healthy subjects, this effect was small and not significant. In the present study, the application of MH did not directly expose the ocular surface to menthol and thus might be considered equivalent to low-dose ointment application. Our results thus suggest that repeated application of MH ameliorated dry eye symptoms.

We would like to emphasize that repeated use of MH over a period of 2 weeks improved TMV and BUT in dry eye patients without the need for administration of any eyedrops or medication. These results indicate that MH application is a potential alternative to eyedrops as a treatment for dry eye patients. Long-term treatment with eyedrops can result in the development of corneal epithelial disorders (toxic keratopathy) and otherwise adversely affect quality of life. Furthermore, dry eye patients with additional ocular diseases such as glaucoma may need to administer multiple types of eyedrops, with the application of those for relief of dry eye symptoms possibly leading to dilution of the drugs contained in the others. Repeated application of MH is thus a candidate novel treatment for individuals with dry eye—including both ADDE and EDE—that would avoid the adverse effects of eyedrop administration. The efficacy of MH for treatment of dry eye thus warrants further evaluation in a multicenter study with a larger number of patients.

## Methods

### Subjects

This study was approved by the Institutional Review Board of Itoh Clinic, and it adhered to the tenets of the Declaration of Helsinki. Written informed consent was obtained from all subjects before examination. Twenty eyes of 20 healthy subjects (10 men and 10 women; mean age ± SD, 34.9 ± 6.8 years) and 36 eyes of 36 patients with dry eye (16 men and 20 women; mean age ± SD, 30.4 ± 5.7 years) who attended Itoh Clinic between March and December 2015 were enrolled in the study (Table 3). Dry eye was diagnosed on the basis of criteria proposed by the Dry Eye Research Group in Japan^28^: (1) the presence of dry eye symptoms, (2) abnormal tear production as determined by Schirmer’s test (≤5 mm after 5 min) or abnormal tear film stability as determined by measurement of tear film BUT (≤5 s), and (3) the presence of conjunctival and corneal epithelial damage as evidenced by a fluorescein staining score of ≥3. The fulfillment of at least two of these three criteria was necessary for a diagnosis of dry eye^29^. Exclusion criteria for all participants included ocular allergies and a history of eye surgery. The healthy subjects had no ocular conditions other than cataract or refractive error. Data were obtained from the left eye of each subject unless this eye did not meet the enrollment criteria, in which case data were obtained from the right eye. One of the 36 patients with dry eye did not undergo single or repeat device application, with the result that the number of such subjects (eyes) was 35 for each protocol.

### Protocols

The effects of two types of disposable eyelid-warming steamer (DELW)^18^ that either contained menthol (MH) (Hogushite-Shaki; Kao, Tokyo, Japan) or did not (HO) (Jo-ki de Hot Eye Mask, Kao) were first evaluated after a single application for 10 min. The two devices were tested on different days with an interval of at least 7 days between each test. The effects of repeated application of MH or HO were then determined after their use for 10 min twice a day for 2 weeks. DELW is an eye mask–shaped device that contains iron powder and water and which provides moist heat through the chemical reaction of iron, water, and atmospheric oxygen when its package is opened. Menthol is volatilized from MH with heat and steam, with the amount of volatilized menthol being ~0.02 mg over 10 min.

### Examinations

For the single-use evaluation, examinations were performed sequentially both before (at least 1 day) and immediately after each device application as follows: (1) DEQS^30^ was determined for assessment of ocular symptoms. (2) TMV was measured over 5 s by strip meniscometry (Echo Electricity, Tokyo, Japan)^31^. (3) Tear film BUT was measured three times consecutively after the instillation of 1 μl of 1% fluorescein with a micropipette, and the mean value was calculated. (4) Corneal and conjunctival epithelial damage was scored from 0 to 9 on the basis of fluorescein staining^29^. (5) The morphology of meibomian glands in the upper and lower eyelids was scored (meiboscore) with the use of a noninvasive meibography system (SL-D701 DC4 BG-5; Topcon, Tokyo, Japan)^32^. (6) Digital pressure was applied to the upper tarsus, and the degree of ease with which meibomian secretion was induced was evaluated semiquantitatively^33^. (7) A Schirmer strip (Whatman no. 41; Showa, Tokyo, Japan) was inserted over the lower lid margin (midway between the middle and outer thirds) for 5 min without topical anesthesia, with subjects being asked to close their eyes during the measurement. Room temperature (25.3° ± 1.2 °C) and humidity (41.2 ± 6.3%) were maintained relatively constant for all examinations. For the repeated-use evaluation, all of the examinations described above were performed both before (at least 1 day) the initial device application as well as during the daytime at least 8 h (but <16 h) after the final application (in order to avoid immediate effects of warming).

### Statistical analysis

Data are presented as means ± s.d. Changes in TMV and BUT between before and after device application were analyzed with Student’s paired *t* test, whereas those in meibum grade and fluorescein staining score were assessed with Wilcoxon’s signed rank test. A *P* value of <0.05 was considered statistically significant.

## Additional Information

**How to cite this article**: Arita, R. *et al*. Effects of a warm compress containing menthol on the tear film in healthy subjects and dry eye patients. *Sci. Rep.* **7**, 45848; doi: 10.1038/srep45848 (2017).

**Publisher's note:** Springer Nature remains neutral with regard to jurisdictional claims in published maps and institutional affiliations.

## Figures and Tables

**Table 1:** Changes in main outcome measures between before and immediately after single application of the heat-only (HO) or menthol-containing (MH) eyelid-warming devices in healthy subjects (*n* = 20) and dry eye patients (*n* = 35).

Parameter	Subjects	Device	Before	After	*P* value
TMV (mm)	Healthy	HO	5.0 ± 1.6	4.5 ± 1.9	0.118
		MH	4.5 ± 1.6	6.9 ± 2.2	8.6 × 10^−5^
	Dry eye	HO	2.7 ± 1.8	2.1 ± 1.3	0.038
		MH	2.5 ± 1.6	4.8 ± 2.6	1.3 × 10^−5^
BUT (s)	Healthy	HO	4.2 ± 1.9	4.9 ± 2.9	0.204
		MH	4.1 ± 1.6	6.1 ± 2.4	0.006
	Dry eye	HO	2.4 ± 1.2	2.5 ± 1.4	0.767
		MH	2.3 ± 1.3	3.4 ± 2.0	0.002
Meibum grade (0–3)	Healthy	HO	0.3 ± 0.6	0.1 ± 0.2	0.025
		MH	0.2 ± 0.4	0	0.046
	Dry eye	HO	0.14 ± 0.36	0.03 ± 0.17	0.046
		MH	0.23 ± 0.43	0	0.005
Fluorescein staining score (0–9)	Healthy	HO	0.2 ± 0.4	0.2 ± 0.4	0.317
		MH	0.3 ± 0.5	0.3 ± 0.5	1
	Dry eye	HO	1.5 ± 1.6	1.7 ± 1.4	0.385
		MH	1.7 ± 1.3	1.8 ± 1.4	0.420

Data are means ± s.d. TMV, tear meniscus volume; BUT, tear film breakup time.

**Table 2:** Changes in main outcome measures between before and at least 8 h after repeated application of the heat-only (HO) or menthol-containing (MH) eyelid-warming devices in healthy subjects (*n* = 20) and dry eye patients (*n* = 35).

Parameter	Subjects	Device	Before	After	*P* value
DEQS	Healthy	HO	NE	NE	
		MH	2.4 ± 2.5	4.2 ± 6.1	0.139
	Dry eye	HO	49.7 ± 18.1	24.2 ± 11.0	0.001
		MH	41.0 ± 18.4	23.2 ± 17.2	9.7 × 10^−4^
TMV (mm)	Healthy	HO	4.3 ± 0.7	4.0 ± 0.7	0.080
		MH	4.5 ± 1.6	6.6 ± 3.2	0.004
	Dry eye	HO	3.2 ± 1.4	3.6 ± 2.0	0.555
		MH	2.2 ± 1.5	4.0 ± 2.1	1.7 × 10^−4^
BUT (s)	Healthy	HO	4.4 ± 1.0	4.9 ± 0.8	0.170
		MH	4.1 ± 1.6	6.1 ± 3.3	0.037
	Dry eye	HO	2.7 ± 1.0	2.0 ± 1.1	0.152
		MH	2.2 ± 1.3	3.5 ± 1.6	0.010
Meibum grade (0–3)	Healthy	HO	0.2 ± 0.4	0	0.170
		MH	0.2 ± 0.4	0	0.046
	Dry eye	HO	0.10 ± 0.32	0	0.317
		MH	0.28 ± 0.46	0.12 ± 0.33	0.102
Fluorescein staining score (0–9)	Healthy	HO	0.3 ± 0.5	0.2 ± 0.4	0.350
		MH	0.3 ± 0.5	0.4 ± 0.8	0.739
	Dry eye	HO	1.7 ± 1.3	1.2 ± 1.0	0.197
		MH	1.6 ± 1.4	1.2 ± 1.2	0.114

Data are means ± s.d. DEQS, Dry Eye–Related Quality-of-Life Score; TMV, tear meniscus volume; BUT, tear film breakup time; NE, not evaluated.

**Table 3:** Baseline characteristics of the study participants.

Characteristic	Healthy subjects (10 men, 10 women)	Dry eye patients (16 men, 20 women)
Age (years)	34.9 ± 6.8	30.4 ± 5.7
DEQS (0–100)	2.4 ± 2.5	43.9 ± 18.4
TMV (mm)	5.1 ± 1.7	2.8 ± 1.5
BUT (s)	5.3 ± 2.2	2.4 ± 1.4
Fluorescein staining score (0–9)	0.3 ± 0.4	1.4 ± 1.1
Meibum grade (0–3)	0.23 ± 0.48	0.22 ± 0.42
Meiboscore (0–6)	1.9 ± 1.0	2.4 ± 1.3
Schirmer test value (mm)	14.9 ± 12.0	5.2 ± 5.3

DEQS, Dry Eye–Related Quality-of-Life Score; TMV, tear meniscus volume; BUT, tear film breakup time.
